# Recruitment, Enrollment, and Retention: Creative Techniques to Overcome Obstacles

**DOI:** 10.1093/geroni/igab046.806

**Published:** 2021-12-17

**Authors:** Justine Sefcik, Darina Petrovsky

## Abstract

The process of recruiting, enrolling, and retaining older adults in research studies has been challenging, even prior to the COVID-19 pandemic. This symposium presents research conducted and lessons learned on recruiting, enrolling, and retaining older adults, including those with cognitive impairment. Insights are provided on what techniques are most beneficial for improving rates of research participation, spanning time prior to and during the pandemic. The first presentation reports on qualitative perspectives of persons living with dementia and their caregivers as to what helped them decide to enroll into a clinical trial together. The second presentation speaks to how variations in incentive payment allocations played a role in consent decisions of patients with amnestic mild cognitive impairment and their study partners. The third presentation discusses the effectiveness of an adapted framework and strategies to increase the recruitment and retention of older Latinos with Alzheimer’s Disease and Related Dementias (ADRD) into a clinical trial. The fourth presentation shares techniques for recruiting older adults for a survey study during the pandemic. The fifth presentation defines challenges during a longitudinal study when the pandemic and other natural disasters occurred and strategies for success. Taken together, these presentations will inform researchers on techniques that could be used to improve recruitment, enrollment, and retention of older adults in clinical research.

